# Cash, Card or Smartphone: The Neural Correlates of Payment Methods

**DOI:** 10.3389/fnins.2019.01188

**Published:** 2019-11-05

**Authors:** Maria Gabriella Ceravolo, Mara Fabri, Lucrezia Fattobene, Gabriele Polonara, GianMario Raggetti

**Affiliations:** ^1^Department of Experimental and Clinical Medicine, School of Medicine, Marche Polytechnic University, Ancona, Italy; ^2^Centre for Health Care Management, School of Medicine, Marche Polytechnic University, Ancona, Italy; ^3^Department of Management and Law, School of Economics, University of Rome Tor Vergata, Rome, Italy; ^4^Department of Economics, Università Lum Jean Monnet, Casamassima, Italy; ^5^Department of Odontostomatologic and Specialized Clinical Sciences, School of Medicine, Marche Polytechnic University, Ancona, Italy

**Keywords:** neuroeconomics, payment methods, consumer behavior, financial services, fMRI

## Abstract

Information technology innovations have pushed toward the digitalization of payments. We carried out an exploratory study to understand if and how brain activity can be modulated by the method of payment (cash, card, and smartphone) or the amount of paid money (10€, 50€, 150€), or both. Sixteen healthy, right-handed, volunteers (eight females) underwent a fMRI session, during which 3 runs were presented with block-designed protocol. Each 5-min run was composed of a standard sequence of 12 *videoclips*, each lasting 12 s and alternated with 12s-rest periods, displaying a human hand paying, each time, through a different method. When contrasting the BOLD signal change by payment method, a greater activation of the parietal cortex (BA40) and right insula (INS) was observed during the exposure of subjects to videoclips showing payments with *cash* than with either *card* or *smartphone*, with any amount of money. A significant greater activation of the right BA40 was observed with 150€ than 50€ and 10€, as well as of the right INS and posterior cingulate cortex (PCC) with 150€ than with 10€, only in the *cash* condition. This pilot study indicates that cash enhances the salience and negative affective valence of parting with money, as suggested by the greater activity of areas processing the perceived utility of motor behavior (e.g., the parietal cortex), and the individual emotional involvement (e.g., INS). By highlighting that cash payment could represent a stronger self-regulating tool, these findings could be relevant for those interested in regulating compulsive shopping or digital gambling.

## Introduction

For millennia, the use of money has stimulated the sensory system and influenced the individual habits to save or spend coins and banknotes. Shape, weight, solidity, and material stimulate vision, proprioception and touch; the jingle of coins or the swooshing of banknotes activate the hearing while the smell of paper money stimulates the olfactory system. The recent and still ongoing technological and financial revolution has introduced electronic money, accelerating the transition toward a so-called cash-less society, providing, through dematerialized money, different sources of sensory stimuli with respect to the traditional ones delivered for centuries by paper and metallic currency. In fact, such dematerialized money likely mitigates the emotions linked to the reward/regret to accumulate/pay money, thus affecting individual spending behavior and attracting the attention of researchers in different disciplines as economics, finance, and psychology (Runnemark et al., 2015).

The hypothesis that money format plays a role in modulating individual mental representation and value perception has been investigated by comparing the influence of cash, pre-paid, debit and credit card, check, and electronic transfer, on subjective transactional awareness (See-To and Ngai, 2019), spending behavior (Thomas et al., 2011; Meyll and Walter, 2019), willingness-to-pay (Falk et al., 2016), perception of product ownership (Kamleitner and Erki, 2013), memory about purchase (Soman, 2001; Soster et al., 2014) and on the ability to remember the emotions related with previous payments (Srivastava and Raghubir, 2002). The hedonic benefits derived from purchased goods and services seem lower when cash rather than card is used, as the former is expected to trigger the so called “pain of payment,” increasing the salience of the amount paid (Prelec and Loewenstein, 1998; Raghubir and Srivastava, 2008).

Interestingly, while different disciplines are devoting attention to understand the influence of payment methods on decision making, addressing the need for an interdisciplinary approach, no studies so far have applied neuroimaging approaches to understand if physical and digital methods of payment are processed differently by the brain. We conducted a functional magnetic resonance imaging (fMRI) experiment to investigate the neural correlates associated with the observation of payment transactions realized through different methods. We exploited the mirror neuron system (MNS) theory, according to which the observation of others’ actions and emotions recruits different brain networks which transform the sensory information concerning others into one’s own motor and visceromotor representations of those actions and emotions (Rizzolatti and Craighero, 2004). fMRI studies report that MNS neurons discharge not only when a subject performs a specific action, but also when he sees the action made by others (Iacoboni et al., 1999; Grezes et al., 2003; Iacoboni and Mazziotta, 2007); according to a few studies, MNS activity increases with familiarity with motor actions, based on how often they are performed or observed (Calvo-Merino et al., 2006; Plata Bello et al., 2013).

On these premises, we conducted a pilot fMRI analysis of BOLD signal change induced by the exposure to videoclips where different methods of payment were used, in different amounts, and we aimed to test the following hypotheses:

H_1_: the average BOLD signal variations during exposure to active stimuli with respect to the rest phase are modulated by the method of payment.
H_2_: the average BOLD signal variations during exposure to active stimuli with respect to the rest phase are modulated by the amount of the payment.

## Materials and Methods

### Participants

Sixteen healthy right-handed volunteers aged 22 to 30 years (mean 27.1 ± 2.4; 8 females) were studied. We administered a questionnaire to participants in order to collect information about their financial attitudes and shopping behavior. Our sample was homogenous in that most of the participants: (a) declared an annual income comprised between 12000€ and 24000€, (b) had at least one bank account, (c) used to withdraw money from ATM, at least once a week, by an amount ranging (d) between 50€ and 150€. They also: (e) use the card method payment in 50% of cases, (f) own a smartphone, but (g) have not enabled payments through it, (h) prefer cash for payments under 50€, while (i) card for payment over 50€. Finally, they (j) rarely buy expensive goods or services, (k) avoid payment in instalments, and (l) consider themselves as rational buyers.

### Stimuli

A stimulation block designed paradigm was used. During scanning, participants underwent three functional runs (one for each amount to be payed, i.e., 10€, 50€, 150€), during which they observed three videoclips, of 12 s (s) each, that varied for the method of payment displayed (*cash, card, smartphone*). Each functional run started with a rest period of 12 s. As mirror neurons are best triggered by visual stimuli displaying an interaction between an object and an effector, we chose to display the act of paying, not the payment instrument itself. Cash payment videos showed a hand leaving the banknote on a white table where another hand collected it. Card payment videos showed a hand inserting a card in the Point of Sale (POS). Finally, smartphone payment videos showed a hand placing a smartphone near the POS, exploiting the Near Field Communication (NFC) technology. Each videoclip was alternated to a 12 s rest period. The 3 videoclips and the subsequent rest periods were repeated four times, up to a total 300 s scanning time. Each block started with a 2 s frame indicating the amount of money that would have been displayed in the subsequent frames (10€, 50€, 150€) and finished with a 2 s frame asserting “Paid,” thus indicating that the operation was concluded. In the *cash* condition, the transaction magnitude was conveyed through the different banknotes, while in both the *card* and *smartphone* conditions it was made discernible by the digits displayed on the POS. The sequence of videoclips was randomized by method of payment and amount to be paid, ensuring that all subjects were exposed to the same number and content of independent stimuli.

In order to standardize the visual stimuli, we reduced the details concerning the human presence to a minimum: the video just displayed one hand without body hair, jewels, nail polish, or any other detail that would have helped subjects to identify it as a female or male hand. Moreover, the hand movement was always from the left bottom end to the center of the screen, applying the same trajectory and occupying the same screen space, across all the videoclips; finally, the background was blank, so that videoclips only differed in the method of payment displayed. The technical quality of the videos was controlled with respect to color brightness and image resolution and kept stable across stimuli.

### Imaging Protocol – fMRI

Subjects were placed in a 1.5 Tesla scanner (Signa LX NV/i, General Electric Medical System, Milwaukee, WI, United States) equipped with 50 mT/m gradients with the head restrained in a circularly polarized head coil. They were instructed to find a comfortable position, to relax, avoiding even minimal movements, and focus attention to the videoclips.

The fMRI experimental procedure consisted of four steps. In the first, an anatomical sagittal localizer (T1 FLAIR, 2D, TR 1675 ms, TE 24 ms, Field of View 30 × 30 cm, slice thickness 5 mm, Matrix 416 × 320, 2 Nex, scan time 1:56 min) was acquired to select the section levels. Twenty contiguous 5-mm-thick axial sections were selected. The second step involved acquisition of a 3D data set (IR Prep Fast SPGR 3-D; TR 15.2 ms, TE 6.9 ms, TI 500 ms, Flip Angle 15°, FOV 29 × 29 cm, slice thickness 1 mm, matrix 288 × 288, 1 Nex, and scan time 8:20 min).

The third step involved the acquisition of axial anatomical T1 weighted images (TR 1700 ms, TE 24 ms, Field of View 24 × 24 cm, thickness 5 mm, Matrix 256 × 256, 1 Nex, scan time 2:25 min for 20 images) on which the functional activations were overlaid. The fourth step involved fMRI acquisitions in the same axial planes with single-shot T2^∗^-weighted gradient-echo EPI sequences (TR 3000 ms, TE 60 ms, Flip Angle 90°, Field of View 24 × 24 cm, Matrix 64 × 64, 1 Nex, scan time 5:12 min) to obtain, during the stimulation cycle, 2000 axial functional images (100/section, 1 image/3 s) from the 20 contiguous 5-mm-thick axial sections selected under step 1.

### Data Analysis

#### Functional fMRI

**FIGURE 1:**
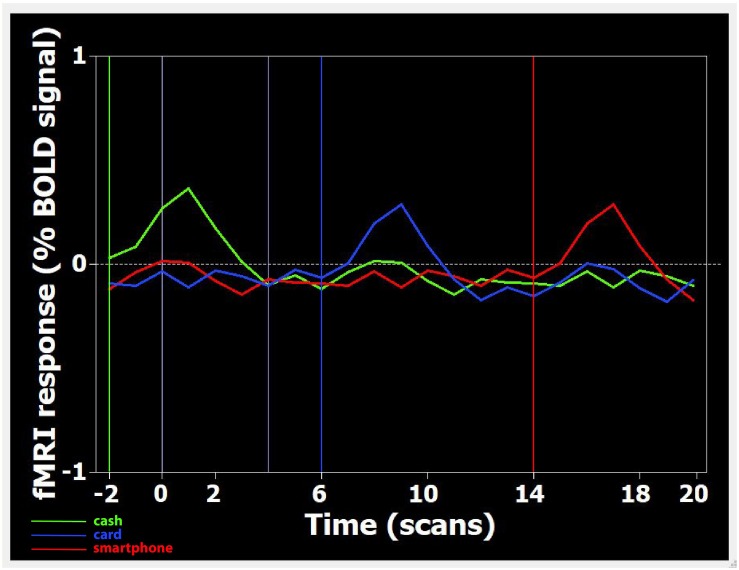
Time course of BOLD signal changes in the parietal area, during the presentation of one Cash-Rest-Card-Rest-Smartphone stimulus sequence in a time window of 60 s. On the *X*-axis, the different digits refer to the number of acquired volumes (1 volume = 3 s). The BOLD signal change is plotted starting from two volumes (i.e., 6 s) before the first stimulation period up to 20 volumes (60 s) after. Since during functional run processing the first 2 volumes are skipped (see section “Materials and Methods”), video onset is actually at –2 volumes (i.e., 6 s before 0). Each colored curved line represents the average BOLD signal change detected, in the whole sample, during the visualization of Cash (green), Card (blue), and Smartphone (red) payment of 150 Euro. Each colored vertical line indicates the onset of footage displaying the relative payment method.

After the experimental session, the images acquired were transferred to a Unix workstation (General Electric Advantage Windows 4.2), and then to a personal computer. Data were analyzed with the BrainVoyager QX software 2.3 version (BV QX; Brain Innovation, Maastricht, Netherlands). The first two images (two volumes, i.e., 6 s) of each functional series were discarded to take into account the period of signal intensity variation occurring due to progressive saturation. Data from each subject were pre-processed to remove noise and artifacts. Pre-processing of functional scans included 3D motion correction: hence, as performed in previous studies (Fabri et al., 2006; Mascioli et al., 2015), subjects presenting movement artifacts (i.e., translation/rotation movements >3 voxels, as automatically detected by the software) were discarded. Cubic spline interpolation was set as the default method in the data preprocessing dialog in BrainVoyager for the slice timing correction. No spatial smoothing was applied to avoid the reduction of spatial resolution assumed to be provoked by this kind of pre-processing (Coalson et al., 2018). The functional images of each subject were overlaid on the 2D anatomical images and co-registered into their 3D data sets through trilinear interpolation. The 2D anatomical images were FSE T1 FLAIR (TE 24, TR 1700, band width 20.83, FOV 24 × 24, matrix 256 × 256, 1 nex, 5 mm interleaved). Data were then transformed into Talairach space (Talairach and Turnoux, 1988). Statistical analysis was performed on data from each subject using the general linear model (GLM). This model aims to predict the variation of a dependent variable (the fMRI time course) in terms of linear combination. To respect the hemodynamic delay, the predictor time course was convolved with a standard hemodynamic response function (HRF). The whole brain was inspected. The activation foci to be studied were selected when clusters were at least of 10 active voxels. When the signal increase in the activation foci was correlated temporally with the stimulation pattern (*p* < 0.05), and was significantly different from the baseline, activation was assumed to be evoked by the stimulation. Figure 1 shows an example of the time course of BOLD signal changes in the parietal area: a direct correlation with videos displaying the three different payment methods is observable.

##### Multi-subject analysis

For multi-subject analysis, data collected from individuals studied in identical experimental conditions were processed as a single data file. To this purpose, a multi-study design matrix (MDM) file was created using GLM. The MDM file was created using a selected functional data file for each run of each subject, which was included in the analysis. The analyzed blocks were of the same duration, i.e., 12 s video/12 s rest. The 12 s stimulus also included the 2 s frames introducing the amount to be paid and the feedback about the payment. The onset vector of the first event (the 12 s video), occurring after a rest interval of 12 s, was modeled as a square function starting at 12 s and ending at 23 s. The other onset vectors were built accordingly. Talairach coordinates of activated cortical areas were then analyzed. Contrasts were therefore calculated for the *cash* stimulus against *card* and against *smartphone*, and for the *card* stimulus against *smartphone*, separately for each amount of money (10€, 50€, 150€). In addition, contrasts were calculated, separately for each payment method, for the highest amount (150€) against the middle (50€) and the lowest (10€), and for 50€ against 10€. The effects were thresholded at *p* < 0.05 and corrected for False Discovery Rate (FDR). As this was an exploratory study we decided to record also all non-FDR corrected effects. The extent threshold employed was a 10 voxel cluster.

#### Ethical Review

This study was carried out in accordance with the recommendations of the Institutional Review Board of the University of Rome Tor Vergata. All subjects gave written informed consent in accordance with the Declaration of Helsinki and Oviedo Convention, after receiving an explanation of the procedure and aims of the study, which was approved by the quoted ethics committee.

## Results

Data from two out of 16 cases were discarded, due to the presence of movement artifacts. The following results refer to the analysis of the remaining 14 subjects (six females).

**FIGURE 2:**
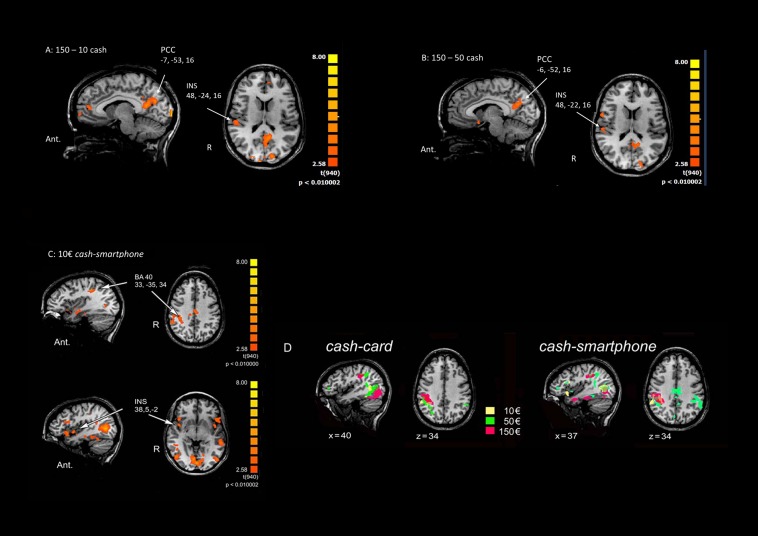
Main effects of contrasting methods of payment and payment amounts. **(A)** 150€ vs. 10€ in the *Cash* condition. **(B)** 150€ vs 50€ in the *Cash* condition. **(C)** *Cash* vs *Smartphone* contrast in the 10€ condition. The Talairach coordinates refer to the activated foci pointed by arrows. Ant.: anterior; R: right. **(D)** Cortical activation in the right parietal cortex in *Cash* vs *Card* (left) and the *Cash* vs *Smartphone* (right) contrasts in the three conditions (10€: yellow; 50€: green; 150€: red).

Multi-subject analysis revealed a modulation of the BOLD signal change by the visual stimulus compared to rest state, with a significant effect for the primary and secondary visual cortex, parietal cortex (BA40), insula (INS), anterior, and posterior cingulate cortex (PCC) and frontal areas (BA6 and BA10) (Table 1). An increasing activation of the BA40, INS and PCC was observed with the exposition to growing amounts of payment, only in the *cash* condition (Figures 2A,B).

**TABLE 1:** Talairach coordinates, percent of signal increase and activated voxels in the cortical activated areas as obtained from multisubjects analysis.

**Condition**		**150€ ^∗∗^**				**50€ ^∗∗^**					**10€^∗^**					
**Contrast**	**Area**	***X***	***Y***	***Z***	**% var. BOLD**	**Voxels**	***X***	***Y***	***Z***	**% var. BOLD**	**Voxels**	***X***	***Y***	***Z***	**% var. BOLD**	**Voxels**
**(A) Cash vs Rest**	**BA 40**	**34**	−**38**	**35**	**0.33**	**618**	**33**	−**45**	**35**	**0.36**	**763**	**33**	−**45**	**34**	**0.19**	**645**
	**INS**	**45**	−**28**	**18**	**0.33**	**56**	**36**	−**33**	**18**	**0.11**	**139**	**34**	−**31**	**17**	**0.15**	**171**
	**PCC^§^**	−**6**	**–52**	**14**	**0.29**	**148**	**–**	**–**	**–**	**–**	**–**	**–**	**–**	**–**	**–**	**–**
**(B) Cash vs Card**	**BA40**	34	−37	34	0.36	798	33	−35	34	0.36	112	37	−45	34	0.23	508
	**INS**	49	−29	21	0.21	86	41	13	1	0.27	106	44	5	14	0.28	178
**(C) Cash vs Smartphone**	**BA40**	34	−39	35	0.38	705	34	−33	35	0.21	426	33	−35	34	0.30	592
	**INS**	45	−24	23	0.55	203	42	−25	17	0.13	423	38	5	−2	0.30	236

**Condition**	**Cash**															

**Contrast**	**Area**	***X***	***Y***	***Z***	**% var. BOLD**	**Voxels**										

**(D) 150€ – 10€**	**BA 40**	40	−35	35	0.32	183										
	**INS**	49	−25	17	0.16	438										
	**PCC (BA29)**	−3	−52	11	0.44	591										
**(E) 150€ – 50€^§^**	**BA 40**	34	−38	36	0.40	136										
	**INS**	50	−23	20	0.30	462										
	**PCC (BA 29)**	−2	−47	12	0.45	346										

*(A) Activations from the contrast Cash-Rest in the 150€, 50€, and 10€ conditions. (B) Activations from the contrast Cash-Card in the 150€, 50€, and 10€ conditions. (C) Activations from the contrast Cash-Smartphone in the 150€, 50€, and 10€ conditions. (D) Activations from the contrast 150€-10€ in the Cash condition. (E) Activations from the contrast 150€-50€ in the Cash condition. ^∗^p < 0.05; ^∗∗^p < 0.01. For clarity purposes results related to the highest and lowest amount of money are reported. BA = Brodmann area; INS = insula; PCC = posterior cingulate cortex. Statistics were FDR corrected, except where specified; § = Non-FDR, p < 0.05.*

When contrasting the BOLD signal change by payment method, a greater activation of the parietal cortex (*p* < 0.01) and right INS (*p* < 0.05) was observed during the exposure of subjects to videoclips showing payments with *cash* than with either *card* or *smartphone*, with any amount of money (Table 1). The analysis performed to measure the effect of using different methods of payment and amounts of money revealed a significant greater activation of the right BA40 with 150€ than 50€ and 10€ (*p* < 0.005), as well as of the right INS (*p* < 0.05) and bilateral PCC (*p* < 0.05 non-FDR corrected) with 150€ than with 10€, only in the *cash* condition (Table 1). The greatest modulation of right INS activation was observed when contrasting *cash* with *smartphone* in the 10€condition (Figure 2C). Figure 2D shows that the greater the amount the larger the right parietal cortex activation when contrasting cash with any digital method of payment. No significant BOLD signal changes were detected in the *card* and *smartphone* conditions with any amount of payment.

## Discussion

This pilot fMRI investigation was carried out in people watching different methods of payment used in a simulated purchase scenario. We found that when a subject observes the action of a cash payment, a stronger activation of brain areas, involved in computation tasks and processing of aversive stimuli, is detected, respect to when the same individual is watching a payment by card, or by smartphone. This effect is progressively enhanced for growing amounts and seems congruent with the millennial habit of the human brain to consider cash payment as an objective reduction of personal monetary wealth.

We observed a significant activation of the parietal cortex, and, particularly, of the inferior parietal lobule (IPL). This area has been associated with the translation of value attribution in a behavioral response. More specifically, values modulate decisions in the parietal cortex, which associates sensory information with motor commands, guiding attention. According to the literature (see, for example, Cohen and Andersen, 2002; Padoa-Schioppa and Assad, 2006; Glimcher and Fehr, 2013), sensory-motor processes and attention incorporate the value of alternative interpretations of sensory signals that are used to guide behavior. Therefore, IPL associates sensation and potential behavior according to the expected value of a series of possible behavioral responses (Hansen et al., 2012). Kahnt et al. (2014) showed that the inferior parietal cortex encodes the importance of cues and is involved not only in shifting attention and accumulating further information, but also in guiding utility-maximizing behavior. Additionally, activity in IPL is associated to the memory of past results, so that decisions are computed in IPL, based on expected values built upon experience, and associated to a motor command (Wisniewski et al., 2015). Interestingly, the activity of this area is independent of “what” (fuel, food, social factors, etc.) and “how” (i.e., the modality, either visual, tactile or olfactory) (Deaner et al., 2005; Hayden et al., 2007; Klein et al., 2008), since afferent signals to IPL have been already converted in a fungible value in other areas (e.g., the orbitofrontal cortex; see for example Padoa-Schioppa and Assad, 2006; Padoa-Schioppa and Assad, 2007). In this study, subjects were not involved in any motor task or requested to take any decision; however, the coupling between sensory (visual) information about payment amount and the act of paying was conveyed through the videoclip and might have provided the basis for the activation of the IPL. The greater activation of the right parietal cortex observed with *cash* payments, than *card* or *smartphone*, with an effect increasing with the amount of money paid, could signal the relevance of the visual cues in terms of perceived utility of motor behavior. As videoclips just differed in terms of the method used for payment, and the amount displayed on the screen, the perceived utility was likely triggered by the act of paying by physical money, though not by the same gesture aimed at a digital purchase. Payment by cash seems to be perceived by human brain as a significantly more salient stimulus than payment by card or smartphone. It is also expected to elicit more negative reactions than paying through electronic money.

Insula plays a key role in the emotional processes and its activation is generally associated with negative emotions such as fear, anger, disgust, pain. We found a significant greater activation of insula in the *cash* than in *card* or *smartphone* conditions, with the anterior part mostly involved when small amounts were displayed, and the posterior part mostly involved when the highest amounts were shown. Data from electrical stimulation recently provided a functional mapping of insula (Mazzola et al., 2019), highlighting the bipolar organization of such area, with a posterior part assigned to somatosensory functions, and notably to pain perception, and an anterior part assigned to visceral functions. Kuhnen and Knutson (2005) and Smith et al. (2014) described a greater activation of the right aINS when subjects experienced the prediction of money loss. The insula represents also a key node of the “salience network” which detects the most relevant stimuli among the internal and external competing ones (Uddin, 2015, 2017); it has a crucial role in cognition (Chang et al., 2013) since it originates feeling states that in turn determine the salience of competing stimuli, detecting their relevance and marking salient information for further cognitive processing (Uddin, 2015). Cumulating evidence revealed that aINS, together with dorsal anterior cingulate cortex (dACC) activation, is associated with fear and avoidance, and with painful experiences when the subjects imagined (Decety and Grezes, 2006; Jackson et al., 2006; Lamm et al., 2007) or recalled a painful experience (Ogino et al., 2007; Fairhurst et al., 2012), even without actually receiving the stimuli. fMRI studies revealed that when an individual is assessing the gains and losses for a risky financial decision, the aINS and the ventral striatum, as the core components of the risk-related network, were frequently activated (Knutson and Huettel, 2015). In these cases, the aINS activation was closely associated with anticipation of aversive stimulus (Simmons et al., 2006), and its functional connectivity with the dACC would reflect a heightened salience about pain (Wiech et al., 2010). The aINS activation may hence play a critical role in the aversion of losses (Knutson et al., 2007). Recent studies have highlighted a crucial role of the aINS in transferring relevant information to the dorsolateral prefrontal cortex which in turns controls attention and working memory (Menon and Uddin, 2010; Sreenivasan et al., 2014). Therefore, in the present study the involvement of the INS might be related to the higher attention dedicated to process the videoclips displaying cash payment which represented a more salient stimulus, as well as to a higher negative perception of paying through physical money rather than dematerialized money. On the other side, the activation of the posterior INS could refer to its involvement in a network underpinning the detection of potential threats, as described by Canessa et al. (2013). Based on their findings, while the striatum anticipates financial losses through detecting “aversive” prediction errors, the right posterior INS and the centromedial amygdala nuclei likely reflect the output of the anticipatory process and particularly the avoidance of those actions that may result in the negative emotions associated with monetary losses. According to this view, loss aversion could be grounded in a neural mechanism whereby individuals come to avoid actions that may entail aversive outcomes, whose sensory properties are represented in amygdala and posterior INS. In the present study, the activation of the posterior INS in subjects looking at the gesture of paying with cash (rather than digital money) could reflect the anticipation of an aversive outcome that would lead to the avoidance of the action of paying (should the subject be really involved in a monetary transaction). Conversely, the mental representation of paying with card or smartphone, triggered by the videos displaying such actions, is supposedly not relevant enough to engage the activation of this network.

As a non-FDR-corrected effect, an increasing activation of the PCC was found to be related to the amount of payment, only in the cash condition. Studies in humans identified the PCC as an area that encodes the expected subjective value, especially for money, making part of the specific valuation network involved in choices related to monetary rewards and losses (Levy and Glimcher, 2011). In the present study, the modulation of the PCC only by cash payments of different amount seems to confirm that digital money is valued differently from cash and is not salient enough to be processed by the money-specific valuation network.

This study is not without limitations. First, the passive task adopted by our protocol might have caused potential drops in attentional engagement and task attendance by the participants. Second, in decision-making research, relying on simulated decisions rather than real ones is less informative, and previous studies have highlighted the importance to study real choices made through own financial resources (Raggetti et al., 2017). Third, we relied on the MNS paradigm which has recently started to attract economists’ interest (Kirman and Teschl, 2010; Eskenazi et al., 2016; Farmer et al., 2016); anyway, even if widely accepted, we should sound a word of caution when interpreting results since it is surrounded by some skeptical opinions about its working as a dominant network specialized in understanding others’ actions (Heyes, 2010), its functioning in human, its involvement in empathy (Baird et al., 2011), its relation with higher social cognitive functions (Hickok, 2009) and therefore also in its relation with representing financial value. Last, but not least, sample size is not powered enough for conclusive findings. Indeed, it was shaped for a pilot study, to form the basis for higher powered trials aimed at investigating neural correlates of using different methods of payment. Due to the exploratory value of the study, we chose to describe also the small effect observed in the PCC, when contrasting the Cash to the Rest phase, considering the role of this area in encoding the expected subjective value for money.

The “pain of paying” has been largely studied in psychology, economics and finance, by means of traditional investigation techniques as questionnaire and interviews. By applying fMRI, this study could be the first to provide the neurophysiological background of the phenomenon by showing how traditional or digital payment methods trigger the activation of different neural structures. This research supports the view that money format affects the individual perception of value. In fact, not seeing physical money going away reduces the salience of parting with money, and thus the painful sensation associated to the purchase (Soman, 2003). Cash payment is more salient since consumers compute and realize how much is deducted from their available wealth when they pay, while dematerialized money does not provide this automatic feedback to the subject (Runnemark et al., 2015). Several researches have shown buyer difficulties in the ability to control impulsive behavior (He et al., 2018; Sofi and Najar, 2018; Japutra et al., 2019), which in turn might negatively affect individual’s economic conditions and well-being. Impulsive behavior can be detrimental to both own wealth and health and since buyers struggle to regulate their unconscious impulsive behavior, the evidence that cash payment could represent a stronger self-regulating tool could be relevant for those interested in regulating compulsive shopping.

## Data Availability Statement

The datasets generated for this study are available on request to the corresponding author.

## Ethics Statement

The study has been approved by the Local Independent Ethics Committee (IEC) and conducted in full conformity with the current revision of the Declaration of Helsinki and Oviedo Convention. The subjects have provided informed written consent to the study.

## Author Contributions

MC and GR equally contributed to the conception and design of the work. LF and GP contributed to the data collection. MC and MF contributed to the data analysis and interpretation. LF and MC prepared the draft of the manuscript, which was critically revised by GR and finally approved by all the authors.

## Conflict of Interest

The authors declare that the research was conducted in the absence of any commercial or financial relationships that could be construed as a potential conflict of interest.
